# Clustering of chronic kidney disease and cardiovascular risk factors in South-West Nigeria

**DOI:** 10.15171/jnp.2017.33

**Published:** 2017-02-03

**Authors:** Rotimi Oluyombo, Michael Adeyemi Olamoyegun, Olugbenga Edward Ayodele, Patience Olayinka Akinwusi, Adewale Akinsola

**Affiliations:** ^1^Renal Unit, Department of Internal Medicine, Federal Teaching Hospital, Ido-Ekiti, Ekiti State, Nigeria.; ^2^Metabolic and Endocrinology Unit, Department of Internal Medicine, Ladoke Akintola University of Technology Teaching Hospital, Ogbomoso, Oyo State, Nigeria; ^3^Renal Unit, Department of Internal Medicine, Ladoke Akintola University of Technology Teaching Hospital, Ogbomoso, Oyo State, Nigeria.; ^4^Cardiology Unit, College of Health Sciences, Osun state University, Osogbo, Nigeria; ^5^Obafemi Awolowo University Teaching Hospitals, Ile-Ife, Osun State, Nigeria

**Keywords:** Chronic kidney disease, Cardiovascular risk factors, Clustering

## Abstract

**Background:**

There exists a synergy between chronic kidney disease (CKD) and cardiovascular risk factors (CVRFs) with increased morbidity and poor outcomes.

**Objectives:**

Data relating to this clustering in black homogenous populations is scanty. We aim to investigate this relationship in Nigerian communities.

**Patients and Methods:**

It was a cross-sectional observation study from semi-urban communities in South-West Nigeria. We used modified World Health Organization (WHO) questionnaire on chronic diseases (WHO STEPS) to gather information on socio-demographic data, biophysical and clinical characteristics. Biochemical analysis of plasma samples was done.

**Results:**

We analyzed data of 1084 with mean age of 56.3 ± 19.9 years (33.4% female). Prevalence of stage 3 CKD was 14.2% (3a and 3b were 10.3% and 3% respectively). Prevalence of hypertension (systolic and diastolic blood pressure) and low high-density lipoprotein cholesterol (HDL-C) increased as clustering of cardiovascular (CV) risk factors (CVFRs) increased both in CKD and proteinuria (P < 0.05). CKD prevalence increases with number of risk factors. There was an inverse relationship between increasing risk factors and mean estimated glomerular filtration rate (eGFR) (P < 0.05). Clustering at least 2 CVRFs in the population with CKD compared to those without CKD was significantly higher (76.6% vs. 65.1%, OR: 1.8, 95% CI: 1.2-2.6, P = 0.005). Similarly, in a univariate analysis, albuminuria had an increased odds of clustering (69.7% vs. 59.6%, OR: 1.9, 95% CI 0.6-6.2, P = 0.409). Using multivariate logistic analysis, there is significantly increased odds of clustering when eGFR is <45 mL/min/1.73 m2 (OR: 2.66, 95% CI: 1.12-6.32) and microalbuminuria 1.74 (95% CI: 1.10-2.75).

**Conclusions:**

Reduced kidney function and proteinuria significantly clustered with CVRFs. This data suggests that individuals with CV clusters should be screened for CKD or vice versa and they should be considered for prompt management of their CVRFs.

**Implication for health policy/practice/research/medical education:**

The synergy between CKD and CVRFs increased the morbidity and consequently poor outcomes with significant health and socioeconomic impact. These findings show significant clustering of CKD and CVRFs in homogenous black population - a setting where access to health services is majorly out-of-pocket. This is therefore a call for early detection for prevention, treatment (pharmacological and non-pharmacological) of CKD in people with CVRFs and vice versa.

## 1. Background

Chronic kidney disease (CKD) is an important public health concern with worse outcomes especially among blacks (1,2). There exists a synergy between CKD and cardiovascular (CV) risk factors resulting in increased co-morbidities, hospitalizations and mortality (3,4).

Reduced estimated glomerular filtration rate (eGFR) is an independent risk factor for increased incidence of CV diseases (5,6)and is thought to be related to increased concentrations of inflammatory markers, systemic metabolic disorders, loss of night-time blood pressure dipping (7,8) and increasing left ventricular hypertrophy (9). Also, albuminuria is strongly independently associated with CV diseases resulting in increased intima-media thickness, left ventricular remodelling and myocardial ischaemia (10). It is a reflection of endothelial dysfunction and presents as early sign of nephropathy contributing additional prognostic dilemma to reduced eGFR, CV burden and reduced life expectancy. The severity of albuminuria and glomerular function has incremental effects on the poor outcomes of patients with CKD. CV diseases resulting from the interplay of these pathophysiological changes activate early aging of the CV system in a vicious cycle and account for most CKD-related deaths (11).

The increased incidence of CKD and CV disease in individuals of black ethnicity has been established but the interplay between CKD and CV disease has not been well studied. There is paucity of information from Nigeria and other low-income countries in spite of the burden of CKD, risk factors and associated challenges. Resource limited countries, such as Nigeria, would therefore benefit from establishing good preventive and interventional policies if ‘clustering’ of CV disease and its risk factors is associated with CKD.

## 2. Objectives

The aim of this study was therefore to determine the burden of CV risk factors and their clustering with CKD.

## 3. Patients and Methods

### 3.1 Population selection

The data presented is from a cross-sectional study in 10 communities in Ekiti and Osun States both in South-western Nigeria. Ten communities from Ekiti North and Central senatorial districts were chosen based on their proximity to our hospital.

### 3.2. Instruments

The Modified World Health Organization questionnaire on chronic diseases (WHO STEPS) was used to gather information on socio-demographic data, biophysical and clinical characteristics. The instrument was administered by trained health professionals in the language (principally Yoruba) best understood by participants. The study was conducted over a 6- month period (January 2013 and May 2013).

### 3.3 Sample analysis and measurements

Urine samples were checked for proteinuria with COMBI 10 strips (Medi-Test Combi 10 SGL) and those that tested negative were checked for microalbuminuria using semi-quantitative methods of estimation with a Clinitek^R^ analyser (Bayer HealthCare LLC) and Microalbumin 2-1 Combo strip of Teco Diagnostic USA. The latter was used when there was difficulty getting more of the Clinitek reagent strips. Blood samples were taken for serum creatinine estimation using the Jaffe reaction to analyse samples. CKD-MDRD 4-variable formula was used to estimate GFR using serum creatinine determined from blood samples collected. Samples were also analysed for lipids: total cholesterol (TC), high-density lipoprotein cholesterol (HDL-C), low-density lipoprotein cholesterol (LDL-C) and triglycerides (TG). Data regarding CV risk factors such as old age (≥60 years), hypertension, diabetes, elevated TC, HDL-C and LDL-C cigarette smoking, waist circumference (WC) and body mass index (BMI) were obtained. The number of risk factors present was counted and ‘clusters’ grouped if <2, 2, 3 or ≥4 risk factors were present. We further used some of the variables (systolic blood pressure [SBP], HDL-C, TC, smoking, age and gender) to calculate the group averages of pooled cohort equations (PCE) for cohorts with CKD as measured by eGFR <60 ml/min/1.73 m^2^, albuminuria. PCE measured the global CV risk and predict 10-year risk of atherosclerotic CVD (fatal and non-fatal stroke, non-fatal myocardial infarction or coronary death) (12). Elevated risk is reported if PCE was ≥7.5%.

Hypertension was defined as SBP ≥140 mmHg and/or diastolic blood pressure (DBP) ≥90 mm Hg or use of blood pressure medications. Diabetes was defined as fasting blood sugar 126 mg/dL and non-fasting blood sugar ≥200 mg/dL or on medications for diabetes. Methods of measurement of hypertension, blood sugar and indices of anthropometry were previously reported (13). WC is elevated in males if > 102 cm or >88 cmin females. BMI was classified as obese if ≥30 kg/m^2^. Elevated cholesterol was defined as TC >200 mg/dL. HDL-C is low in males if <40 mg/dL and 50 mg/dL in females and LDL-C is high if >130 mg/dL. For the purpose of this study, CVRFs ≥2 were considered as cluster. Chronic Kidney Disease-Epidemiology Collaboration (CKD-EPI) formula was used to estimate kidney function.

### 3.4. Ethical issues

The research followed the tenets of the Declaration of Helsinki. We sought consent in each community through meeting with the community heads majority of who were Royal Fathers before the commencement of screening exercises in each community. Each participant signed consent form before they were admitted into the study. Ethics approval was also received from Ladoke Akintola University of Technology Teaching Hospital’s research and ethics committee.

### 3.5. Data analysis

Data was analysed using SPSS version 20 (SPSS Inc., Chicago IL, USA). Standard descriptive statistics were used to examine baseline demographic characteristics, clinical and laboratory values. Descriptive statistics was used for continuous variables and summarized as mean ± standard deviation (SD). Categorical variables were reported as frequencies and proportions. Chi-square test was used to analyse associations in discrete variables. We examined the association between eGFR and albuminuria as features of CKD and CVRFs. Using univariate analysis, we also examined the relationship between these features of CKD and clusters of CV factors as 2, 3 and ≥4. We then determined the association and interaction effect of CKD as defined by eGFR and/or albuminuria, age and gender from univariate analysis as independent variables and clustering of at least two risk factors as dependent variable using multivariate logistic statistical analysis to obtain odds ratio. Level of significance was *P *< 0.05.

## 4. Results

Complete data was available for 1084 individuals. 362 (33.4%) of individuals were male. Mean age was 56.3± 19.9 years (age range). A total of 569 (52.6%) individuals were ≥60 years of age. Women were older both in the general population (58.08 ± 18.56 vs. 52.67 ± 21.84, *P* = 0.001) and CKD group (66.32 ± 16.0 vs. 54.59 ± 19.97, *P* = 0.001). In Table 1, we presented demographic and clinical characteristics of study participants by making comparison between those with and without CKD as measured by eGFR, those with and without proteinuria, those with and without cluster of CV risk factors and those without CKD or proteinuria with those with both.

**Table 1:** Demographic and clinical characteristics and risk factor clustering among studied population with proteinuria and eGFR ≤60 mL/min/1.73 m^2^

** Proteinuria**						
**Factors**	**No risk factor** **No. (%)**	**1 risk factor** **No. (%)**	**2 risk factors** **No. (%)**	**3 risk factors** **No. (%)**	**≥ 4 risk factors** **No. (%)**	**p-value**
Age ≥60 (years)	0 (0.0)	12 (6.7)	47 (26.4)	67 (37.6)	52 (29.2)	0.001
Female	4 (1.5)	22 (8.5)	81 (31.3)	79 (30.5)	73 (28.2)	0.001
Male	1 (0.8)	19 (15.3)	55 (44.4)	37 (29.8)	12 (9.7)	0.001
Abdominal obesity	0 (0.0)	1 (1.1)	8 (8.5)	27 (28.7)	58 (61.7)	0.001
SBP (mm Hg)	0 (0.0)	2 (1.1)	37 (20.4)	75 (41.4)	67 (37)	0.001
DBP (mm Hg)	0 (0.0)	5 (4.4)	19 (16.7)	45 (39.5)	45 (39.5)	0.001
Diabetes	0 (0.0)	0 (0.0)	2 (7.7)	9 (34.6)	15 (57.7)	0.001
Low HDL-C (mg/dL)	1 (0.3)	19 (6.4)	101 (34)	98 (33)	78 (26.3)	0.001
High TC (mg/dL)	1 (3.7)	2 (7.4)	3 (11.1)	10 (37.0)	11 (40.7)	0.001
High LDL-C (mg/dL)	0 (0.0)	0 (0.0)	0 (0.0)	5 (35.7)	9 (64.3)	0.001
Cigarette smoking	0 (0)	0 (0)	5 (50)	3 (30)	2 (20)	0.727
**eGFR ≤ 60 mL/min/1.73 m** ^2^						
Age ≥60 (years)	0 (0.0)	5 (5.3)	29 (30.5)	36 (37.9)	25 (26.3)	0.634
Female	0 (0)	8 (6.3)	40 (31.7)	38 (30.2)	37 (88.1)	0.408
Male	0 (0.0)	2 (7.1)	8 (28.6)	13 (46.4)	5 (17.9)	0.408
Abdominal obesity	0 (0.0)	0 (0.0)	5 (12.5)	15 (37.5)	20 (50)	0.001
Diabetes	0 (0.0)	0 (0.0)	0 (0.0)	2 (33.3)	4 (66.7)	0.204
SBP (mm Hg)	0 (0.0)	3 (4.5)	7 (10.4)	24 (35.8)	33 (49.3)	0.001
DBP (mm Hg)	0 (0.0)	1 (2.4)	4 (5.1)	14 (15.9)	20 (39.6)	0.001
Low HDL-C (mg/dL)	0 (0.0)	1 (0.9)	33 (28.4)	44 (37.9)	38 (32.8)	0.001
High TC (mg/dL)	0 (0.0)	114.3)	1 (14.3)	1 (14.3)	4 (57.1)	0.119
High LDL-C (mg/dL)	0 (0.0)	0 (0.0)	0 (0.0)	1 (25)	3 (75.0)	0.257
Cigarette smoking	0 (0.0)	0 (0.0)	1 (50)	0 (0)	1 (50)	0.582

Abbreviations: TC, total cholesterol; ; HDL-C, high density lipoprotein-cholesterol; LDL-C, Low density lipoprotein-cholesterol; ACR, albumin creatinine ratio; eGFR, estimated glomerular filtration rate; SBP, systolic blood pressure; DBP, diastolic blood pressure.

Participants with CKD (Table 2), as compared to those without CKD, had the following significant differences; older age, higher waist circumference and underweight (21% vs. 13.2%, *P* = 0.028). The prevalence of obesity as measured by BMI, however, is lower (4.8% vs. 8.3%, *P* = 0.028) than among those without CKD.

**Table 2:** Patterns of gender distribution and clinical characteristics of cohorts with proteinuria, low eGFR (≤60 mL/min/1.73 m^2^) and clustered CVRFs.

**Factors**	** CKD**	** Proteinuria**	**No CKD/proteinuria**	**Clustering of CV risk factors**
Gender (n, %)				
Male	28 (18.2)^a^	133 (32.3)^b^	221 (37.5)^a^	235 (29.3)^a^
Female	126 (81.8)	278 (67.6)	369 (62.5)	588 (70.7)
Diabetes (n, %)	6 (5.0)^b^	26 (7.9)^b^	27 (6.1)^b^	62 (9.7)^a^
High TC (n, %)	7 (4.5)^b^	29 (7.5)^b^	28 (4.8)^b^	58 (7.8)^a^
Low HDL-C (n, %)	118 (75.8)^b^	299 (77.3)^b^	418 (71.0)^a^	602 (81.4)^a^
High LDL-C (n,%)	4 (2.6)^b^	16 (4.1)^b^	16 (2.7)^b^	37 (5.0)^a^
BMI classes (%)				
Normal	50 (47.6)^a^	143 (54.8)^b^	225 (59.7)^b^	296 (52.5)^a^
Overweight	28 (26.7)	56 (21.5)	76 (20.2)	137 (24.3)
Obese	5 (4.8)	19 (7.3)	30 (8.0)	54 (9.6)
Mean age ±SD	66.4±16.1^a^	57.9±19.4^a^	53.6±20.4^a^	64.6±15.7^a^
SBP	140.6±28.3^b^	140.3±27.8^a^	136.2±26.5^a^	148.3±27.4^a^
DBP	80.7±14.0^b^	81.1±14.6^a^	78.7±12.8^a^	84.0±14.0^a^
Waist circumference	85.2±11.4^a^	83.2±11.6^a^	83.2±11.8^b^	86.5±12.2^a^
Mean BMI	23.0±5.5^b^	22.5±5.1^b^	22.7±4.5^b^	23.5±5.5^a^
Mean blood sugar	98.3±34.1^b^	103.8±36.4^a^	97.7±24.9^b^	101.9±33.5^a^
Median ACR	20^b^	40^a^	15^a^	20^a^
Mean eGFR	48.3±9.8^a^	88.1±26.5^b^	95.3±29.0^a^	83.4±27.9^a^
Cigarette smoking	2 (1.4)^b^	10 (2.6)^b^	5 (1.2)^a^	11 (2.4)^b^

Abbreviations: TC, total cholesterol; BMI, body mass index; HDL-C, high density lipoprotein-cholesterol; LDL-C, Low density lipoprotein-cholesterol; ACR, albumin creatinine ratio; eGFR, estimated glomerular filtration rate; CKD, chronic kidney disease; CVRF, cardiovascular risk factor. ^a^ *P *<**0.05; ^b^*P *>**0.05. Values (means and percentages) in each column are paired to compare with those with and without the factors.

The mean eGFR was significantly lower among those with SBP >140 mm Hg (84.26 ± 23.95 vs. 88.13 ± 26.12 mL/min/1.73 m^2^, *P* = 0.013), DBP >90 mm Hg (82.51 ± 22.22 vs. 87.84 ± 26.12 mL/min/1.73 m^2^, *P* = 0.003), low HDL-C (84.52 ± 24.17 vs. 92.34 ± 27.36 mL/min/1.73 m^2^, *P* = 0.001) and increased waist circumference (89.12 ± 32.42 vs. 94.08 ± 32.71 mL/min/1.73 m^2^, *P* = 0.029) but not BMI than their counterparts with normal values. The mean SBP (141.13 ± 27.83 vs. 137.06 ± 26.93 mm Hg, *P* = 0.021) and DBP (81.57 ± 14.71 vs. 78.97 ± 12.91 mm Hg, *P* = 0.002) were higher among cohorts with proteinuria. Prevalence of hypertension (SBP and DBP) and low HDL-C increased as clustering increased both in CKD and proteinuria (Table 2). Increasing age of participants is associated with increase prevalence of CKD, proteinuria and clustering of CV risk factors.

**Figure 1:**
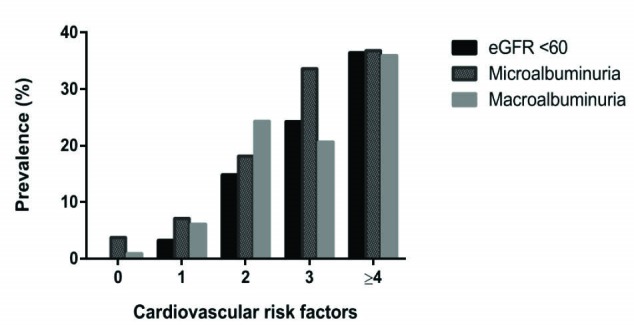
Cardiovascular risk factor clusters, chronic kidney disease and albuminuria.

**Figure 2:**
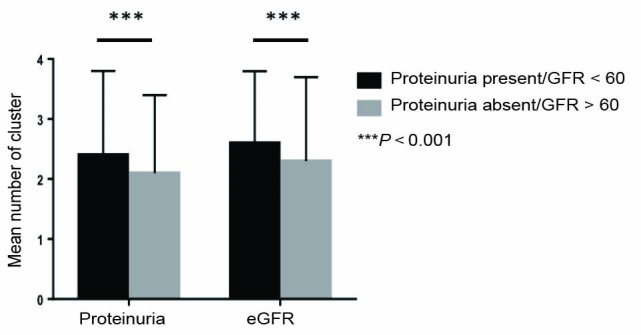
Comparing mean number of cardiovascular risk factors, proteinuria and estimated glomerular filtration rate.

CKD (eGFR of ≤60 mL/min/1.73 m^2^) was seen in 154 (14.2%). CKD stages 3a, b, 4 and 5 were 10.3%, 3%, 0.8% and 0.1% respectively. CKD prevalence significantly (*P* = 0.001) increased with number of risk factors as shown in Figure 1. Of those with CKD, 96 (62.3%) as compared with those without 241 (25.9%), had ≥3 CV risk factors (*P* < 0.05). Prevalence of CKD was non-significantly higher among proteinuric than non-proteinuria cohorts (15.5% vs.12.9%, OR: 1.1, 95% CI: 0.8-1.6, *P* = 0.238). There was a significant inverse relationship between increasing CV factors and mean eGFR (Figure 2). For instance, those who had none compared to those with at least 2 risk factors (98.83 ± 23.61 vs. 85.11 ± 24.61, *P* = 0.001) had higher mean eGFR.

Clustering of at least 2 CV risk factors in the population with CKD compared to those without CKD was significantly higher (76.6% vs. 65.1%, OR: 1.8, 95% CI: 1.2-2.6, *P* = 0.005). In the absence of proteinuria, the odds of clustering of CKD remained high (75% vs. 61.4%, OR: 1.9, 95% CI: 1.16-3.09, *P* = 0.012). We also stratified for gender, men with CKD (75% vs. 55.1%, OR: 2.4, 95% CI: 1.0-5.9, *P* = 0.047) had higher odds of clustering than those without. Females with CKD however, had non-significant higher odds of clustering (77% vs. 70.8%, OR: 1.4, 95% CI: 0.9-2.2, *P* = 0.191).

Microalbuminuria and macroalbuminuria were seen in 194 (17.4%) and 195 (17.5%) individuals respectively. Clusters were more common with the presence of proteinuria than those without (69.7% vs. 59.6%, OR: 1.6, 95% CI: 1.2-1.2.0, *P* = 0.001) in the general population. A total of 201 (52.5%) with albuminuria and 130(18.9%) without albuminuria had ≥3 risk factors (*P* < 0.05). As with eGFR in Figures 2 and 2, both miroalbuminuria and macroalbuminuria increased with increasing number and mean of clusters. Among the cohort with proteinuria without CKD, the odds of clustering are maintained at 1.6 (95% CI: 1.2-2.1, *P* = 0.002). Proteinuria significantly clustered with CV risk factors among males (65.4% vs. 47%, OR: 2.1, 95% CI: 1.4-3.3, *P* = 0.001) participants than females (71.6% vs. 66.5%, OR: 1.3, 95% CI: 0.9-1.7, *P* = 0.152).

**Figure 3:**
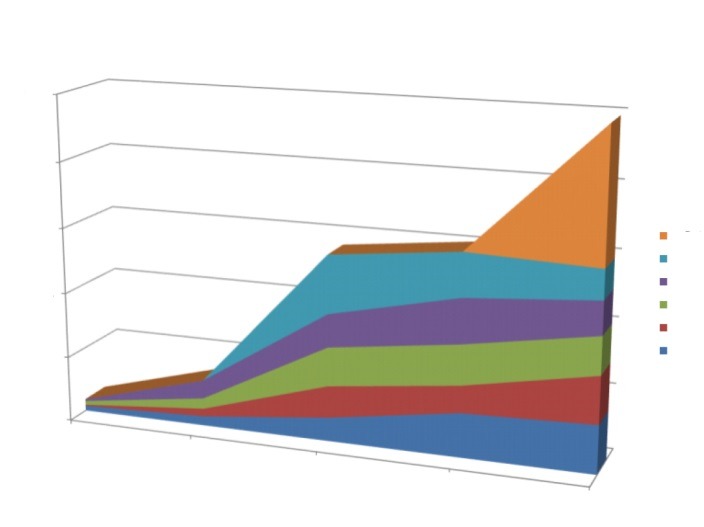
Stage of chronic kidney disease, microalbuminuria and macroalbuminuria in relation to clusters of risk factors.

When CKD was staged, we observed in CKD stage 2 for instance, as risk factors cluster from 0, 1, 2, 3 and 4, the prevalence of CKD significantly increased; 3.1%, 8.2%, 29.6%, 30.6% and 28.6% respectively (Figure 3). The PCE among males with CKD (eGFR) and albuminuria was 14.2% and 7.4% while females had 7.4% and 5.2% respectively.

Using multivariate logistic analysis, we observed an independent significant prediction of having clustering of CV risk factors by albuminuria (OR: 1.75, 95% CI: 1.11-2.77) but not with CKD measured as eGFR <60 mL/min/1.73 m^2^. However, when we analyzed using eGFR <45 mL/min/1.73 m^2^ (CKD 3b) in a similar logistic analysis, we observed an independent increased odds of clustering (Table 3) with CKD 3b and microalbuminuria. We also noticed a positive trend among those with macroalbuminuria.

**Table 3:** The logistic regression of CKD, proteinuria and cluster of risk factors

	** Exp B**	** 95% CI **	**P value**
Female	1.89	1.35-2.64	0.001
Age >60 (y)	24.16	16.35-35.70	0.001
Microalbuminuria	1.74	1.10-2.75	0.017
Macroalbuminuria	1.16	0.76-1.77	0.50
eGFR <45^a^	2.66	1.12-6.32	0.027

Abbreviations: eGFR, estimated glomerular filtration rate; CKD, chronic kidney disease. ^a^ <45 mL/min/1.73 m^2^.

## 5. Discussion

This cross-sectional study from Nigeria has shown an association of CV risk factor clusters with CKD.

Age, abdominal obesity by WC but not BMI, hypertension, and low HDL were significantly associated and clustered with CKD as measured by eGFR and albuminuria. Lemieux et al (14) described that ‘hypertriglyceridaemic waist’ has poor cardio-metabolic profile and renal-related diseases. In Framingham study, low HDL-C co-existing with CKD was found to have a significant predictability of CVD (15).^.^ Interestingly, the prevalence of low HDL-C has been reported to be high among Nigerians (16,17) and there may be need for further exploration of this association since it goes beyond race or region.

The prevalence of CKD using eGFR <60 mL/min/1.73 m^2^ was 14.2%. Other groups have earlier reported similar prevalence in Nigeria (18,19). A review by Stanifer et al also put prevalence of CKD at 12.4% in the urban and 16.9% in rural settlements of Sub-Saharan Africa (20). The high prevalence of proteinuria is ascribed to high prevalence of glomerular diseases and the various reports that blacks are at increased risk of proteinuria than Caucasians with CKD (21,22). There are reports of increased variants of *APOL1* among blacks; Peralta et al (23) observed an increased excretion of albumin among this cohort with two alleles of these genes. Whether this could be generalized remains to be answered. CV profile of proteinuric cohort in our study is different from those with reduced eGFR. Proteinuria compared to reduced eGFR is clustered with majority of the traditional CV risk factors. Proteinuria with reduced eGFR portends increased CV risk factors and poor CV outcomes (24).

Prevalence of clustering of CVRFs is higher among the CKD and proteinuric cohorts than general population studied. Reduced kidney function has increased odds of 1.8 to cluster with other risk factors. Increasing number of risk factors has an inverse relationship with eGFR. In ARIC study, CKD is an independent risk factor for CV diseases (25). Framingham heart study (15) and Muntner et al (26)reported a strong bond between CKD and other CV risk factors but not with CVD. Weiner et al (27), in a meta-analysis of pooled community studies reported CKD as an independent risk factor for CVD and outcomes. They found a significant association between CKD and CVD among black cohort.

We showed that the presence of CKD using GFR <60 mL/min/1.73 m^2^onlyas well as proteinuria alone increased the risk of clustering in a univariate analysis. We further demonstrated that CKD 3b has an independent increased odd of 2.7 with clustering of CVRFs. Likewise, microalbuminuria had increased odds of clustering of 1.7 and macroalbuminuria showing a positive trend. This independence of reduced GFR and albuminuria was also reported by van der Velde et al (28). Fox et al in a meta-analysis of over a million populations reported high risk of CVD in reduced kidney function and proteinuria regardless of the presence of importantly established risk factors (5,6). Risk of clustering of CV factors with moderate to severe CKD (3a and 3b) according to KDIGO was three-fold and two-fold respectively higher than cohort with normal GFR.

Parikh et al (15) in Framingham study, gave a similar report of an association between CKD 3b and CV diseases, but Foster et al (29) failed to establish the consistent association of CKD and CV risk factors. Plausible reasons are the estimating eGFR equation (CKD-EPI in our study) used which has been found to predict CV events more than MDRD equation (30), racial differences in the CV profiles and markers of CKD as shown in our study.

## 6. Conclusions

Reduced kidney function and proteinuria significantly clustered with CVRFs. This data suggests that individuals with CV clusters should be screened for CKD or vice versa and they should be considered for prompt management of their CVRFs. The possible genetic composition cum high prevalence of other CV risk factors such as hypertension in Nigeria and African population in general, may explain this clustering. And if this is the case, it is a call for urgent attention. The ongoing prospective study by the Human Heredity and Health (H3) in Africa Kidney Disease Research Network is expected to unravel some of these genetic conundrum associated with risk of CKD and CV risk factors in black population (31).

## Limitations of the study

In spite of these significant findings, our study is limited by its cross-sectional nature as the causal relationship could not be established. We therefore suggest longitudinal studies in a larger population. Other limitations were the inability to repeat the urine dipstick tests which could have helped to confirm the proteinuria in keeping with NKFKDQI definition of CKD. However, the strength of this study lies in the homogeneity of the black populace in the communities. This would bridge the gap as regards clustering of CVD risk factors in CKD.

## Acknowledgements

We acknowledge the efforts of Dr Rupert Major and Mr Adeleke AA of Nephrology Department, Leicester General Hospital and Chemical Pathology Department, Federal Teaching Hospital, Ido-Ekiti for their help in reviewing and analysing the samples respectively.

## Authors’ contribution

RO, MAO, AA and OEA conceived the idea of this publication. RO, POA, and MAO were involved in data collection. All the authors were involved in literature review, data analysis, review of the article for final publication.

## Conflicts of interest

We declare no conflict of interest in getting this work done and published.

## Funding/Support

We appreciate the partly support of Olorunda Local Government, Osogbo, Osun State, Nigeria.
